# Resolution of alkaloid racemate: a novel microbial approach for the production of enantiopure lupanine via industrial wastewater valorization

**DOI:** 10.1186/s12934-020-01324-1

**Published:** 2020-03-14

**Authors:** Stella Parmaki, Argyro Tsipa, Marlen I. Vasquez, João M. J. Gonçalves, Ioanna Hadjiadamou, Frederico C. Ferreira, Carlos A. M. Afonso, Chrysoulla Drouza, Michalis Koutinas

**Affiliations:** 1 Department of Chemical Engineering, Cyprus University of Technology, 30 Archbishop Kyprianou Str., 3036 Limassol, Cyprus; 2 Civil and Environmental Engineering, University of Cyprus, 75 Kallipoleos Str., 1678 Nicosia, Cyprus; 3 Research Institute for Medicines (iMed. ULisboa), Faculty of Pharmacy, Universidade de Lisboa, Av. Prof. Gama Pinto, 1649-003 Lisbon, Portugal; 4 Department of Chemistry, University of Cyprus, P.O. Box 20537, 1678 Nicosia, Cyprus; 5 Department of Agricultural Sciences, Biotechnology and Food Science, Cyprus University of Technology, 30 Archbishop Kyprianou Str., 3036 Limassol, Cyprus; 6 Institute for Bioengineering and Biosciences, Department of Bioengineering, Instituto Superior Técnico, Universidade de Lisboa, Av. Rovisco Pais, 1049-001 Lisbon, Portugal

**Keywords:** Lupanine, Enantiomers, Enantiomeric excess, Ecotoxicological assessment, *Pseudomonas putida* LPK411, Quantitative real-time PCR, Gene expression

## Abstract

**Background:**

Lupanine is a plant toxin contained in the wastewater of lupine bean processing industries, which could be used for semi-synthesis of various novel high added-value compounds. This paper introduces an environmental friendly process for microbial production of enantiopure lupanine.

**Results:**

Previously isolated *P. putida* LPK411, *R. rhodochrous* LPK211 and *Rhodococcus* sp. LPK311, holding the capacity to utilize lupanine as single carbon source, were employed as biocatalysts for resolution of racemic lupanine. All strains achieved high enantiomeric excess (ee) of L-(−)-lupanine (> 95%), while with the use of LPK411 53% of the initial racemate content was not removed. LPK411 fed with lupanine enantiomers as single substrates achieved 92% of D-(+)-lupanine biodegradation, whereas L-(−)-lupanine was not metabolized. Monitoring the transcriptional kinetics of the *luh* gene in cultures supplemented with the racemate as well as each of the enantiomers supported the enantioselectivity of LPK411 for D-(+)-lupanine biotransformation, while (trans)-6-oxooctahydro-1H-quinolizine-3-carboxylic acid was detected as final biodegradation product from D-(+)-lupanine use. Ecotoxicological assessment demonstrated that lupanine enantiomers were less toxic to *A. fischeri* compared to the racemate exhibiting synergistic interaction.

**Conclusions:**

The biological chiral separation process of lupanine presented here constitutes an eco-friendly and low-cost alternative to widely used chemical methods for chiral separation.

## Background

The advanced performance of pure enantiomers as compared to chiral mixtures has stimulated a growing interest over the past few years in producing single isomer components for the food, fine chemicals, agrochemical and pharmaceutical sectors [1–3]. There is clear evidence of the advantage offered by the preparation of enantiomerically pure drugs, providing the required physiological effect, while reducing the total dose and side-effects caused by the undesired enantiomer [4]. Moreover, the production of enantiopure compounds exhibits strong economic interest and the worldwide sales of single-enantiomer drugs demonstrate a continuous increase [5]. Thus, different approaches have been developed for the production of pure enantiomers, comprising the asymmetric synthesis of a single enantiomer (chiral approach) and the resolution of racemic mixtures (racemic approach) [6].

Lupanine constitutes the major quinolizidine alkaloid present in the wastewater emitted from the manufacturing process of the *L. albus* L. snack [7, 8]. The aforementioned alkaloid has attracted considerable attention in the biotechnological sector and it could be employed as feedstock for semi-synthesis of various novel high added-value compounds based on a series of useful functionalities of its asymmetric structure [9]. However, lupanine is neurotoxic [10] and exists in nature in a racemate of D-(+)-lupanine and L-(−)-lupanine, necessitating the development of separation processes that recover the enantiomers in a pure form and enable the use of each compound for the production of specific fine molecules [11]. Only a few studies have currently achieved chemical resolution of racemic lupanine and conversion of lupanine enantiomers. Przybył and Kubicki, [12] produced optically pure lupanine enantiomers using dibenzoyltartaric acids followed by reduction of L-(−)-lupanine into D-(+)-sparteine, a naturally occurring compound which cannot be easily obtained from natural sources. Moreover, L-(−)-sparteine demonstrates numerous pharmacological properties and chemical applications as ligand or promoter for various asymmetric reactions [13], while the synthesis of D-(+)-sparteine exhibits increased industrial interest [14]. Thus, the production of L-(−)-lupanine from racemic lupanine rich wastewater through bioconversion is expected to open a novel direction for the synthesis of D-(+)-sparteine at a potentially lower cost.

Previous research has reported that different strains are capable of degrading racemic lupanine [15–17]. However, studies on microbial resolution of racemic alkaloids and the metabolic properties of the microorganisms employed are still lacking. Detheridge et al., [18] reported that the first step in lupanine catabolism using *P. putida* Psp-LUP employed hydroxylation of the alkaloid into 17-hydroxylupanine, which was catalysed by lupanine hydroxylase. The specific enzyme is active only when the microorganism is grown on D-(+)-lupanine remaining inactive at the presence of L-(−)-lupanine [19], which enables the selective resolution of the racemic mixture.

This work presents a simple, environmental friendly and safe biotechnological process that reduces the experimentation required for lupanine valorization through chemical methods for the manufacture of enantiopure alkaloids. Resolution of racemic lupanine was targeted through application of *P. putida* LPK411 and two *Rhodococcus* species capable of using lupanine as single carbon substrate. To this end, ee of L-(−)-lupanine was determined during growth on the racemic mixture by all three strains, while the capacity of LPK411 to utilize each of the enantiomers as single carbon substrates was also evaluated. The structure of the end-products formed from D-(+)-lupanine bioconversion was assessed by NMR and the toxicity of each enantiomer was evaluated using *Aliivibrio fischeri*. Additionally, expression from the gene encoding for the first enzyme of the pathway was systematically monitored during biodegradation of racemic lupanine and lupanine enantiomers to assess the enantioselective metabolic properties of LPK411.

## Results and discussion

### Biocatalytic resolution of lupanine enantiomeric mixture

**Fig. 1:**
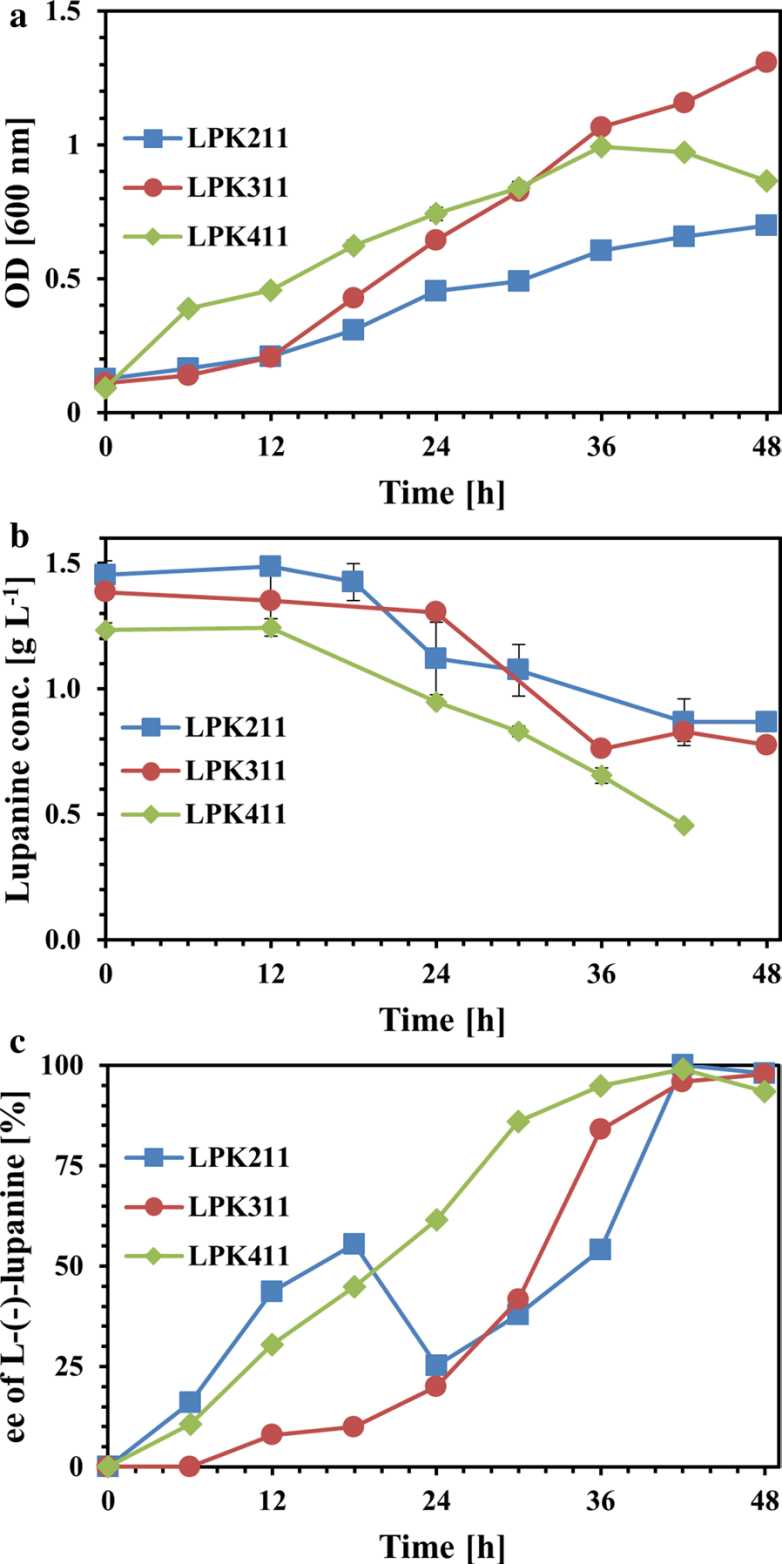
Biocatalytic resolution of racemic lupanine. **a** Biomass growth (OD), **b** lupanine concentration and **c** ee of L-(−)-lupanine (expressed as  %)

Enantiomers comprise molecules that include the same chemical formula as well as identical physical and chemical properties in a chiral environment [3]. However, the biological activity of two enantiomers could be significantly different constituting the cost-effective enantioselective synthesis an important industrial topic [20]. Several processes have been employed for the manufacture of enantiopure compounds. The racemic approach includes processes of extensive industrial use, such as enantioselective liquid–liquid extraction, membrane applications and chromatography. However, employing these methods often requires use of expensive equipment and significant amounts of organic solvents [21]. Thus, biocatalytic resolution of racemic mixtures could be an environmental friendly and low cost alternative for chiral separation. In this study, the enantioselective degradation of racemic lupanine was investigated during fermentation of three microorganisms (*P. putida* LPK411, *R. rhodochrous* LPK211, *Rhodococcus* sp. LPK311), which are capable of utilizing racemic lupanine as single carbon substrate [15]. The results demonstrate that all microorganisms could perform resolution degrading faster D-(+)-lupanine (Fig. 1). LPK211 and LPK311 exhibited high resolution of racemic lupanine at 42 h, which corresponded to 100% and 98% of L-(−)-lupanine ee respectively. Moreover, approximately 60% of the initial racemic lupanine content still remained in the biomedium following 42 h at both cultures demonstrating that a substantial part of L-(−)-lupanine could still be potentially present. However, *P. putida* LPK411 reached L-(−)-lupanine ee of 95% more rapidly (36 h) as compared to *Rhodococcus* strains, which was further increased to 99% at 42 h. The remaining racemic lupanine from LPK411 cultivation at 36 h was 53% highlighting the great potential of the strain for biocatalytic L-(−)-lupanine purification.

The literature has shown that *Pseudomonas* and *Rhodococcus* species are capable of biodegrading enantioselectively various compounds, including aromatic and other organic molecules (Table 1). Ettireddy et al. [22] reported that immobilized cells of *P. putida* (NCIB9494) were capable for resolution of racemic carvedilol within 10 h, accumulating *(S)*-(−)-carvedilol at a concentration of 34.92% and purity that included 94.67% ee. Furthermore, resting cells of *Rhodococcus* sp. CCZU10-1 could remove 44.7% of racemic phenyl methyl sulfoxide, achieving enantioselectivity of *(S)*-phenyl methyl sulfoxide that reached ee > 99.9% [23]. Resolution of racemic mixtures has been also achieved through the use of purified enzymes. Wu et al. [24] demonstrated that ω-transaminase from *P. putida* NBRC 14164 entailed high enantioselectivity to different racemic amines and amino alcohols, including α-methylbenzylamine and 2-amino-2-phenylethanol. The enzyme PpspuC achieved ee > 99.0% of *(R)*-α-methylbenzylamine and *(S)*-2-amino-2-phenylethanol within 4 h and 24 h respectively, while the removal of racemic α-methylbenzylamine was 50.2% and that of racemic 2-amino-2-phenylethanol reached 50.7%. Moreover, the oxidase *R. opacus* L-AAO incorporated wide substrate specificity and it was capable of performing resolution of racemic amino acid mixtures, including leucine and phenylalanine [25]. The results show that the purified enzyme achieved ee > 99.5% for d-phenylalanine and ee > 99.2% for d-leucine within 24 h, demonstrating that d-leucine and d-phenylalanine concentrations were constant during the process. However, Mesnard et al. [26] reported that in the presence of racemic nicotine, *Nicotiana plumbaginifolia* could degrade more rapidly *(R)*-nicotine as compared to the natural isomer *(S)*-nicotine. Thus far, studies have mainly focused on application of chemical methods for purification of lupanine enantiomers from the racemic mixture. Specifically, separation of the enantiomers could be achieved using solvents such as (+)-dibenzoyltartaric acid and ethanol in several steps [12, 13]. The findings of the present study suggest that a two stage process could be developed, including microbial resolution of racemic lupanine to produce enantiopure L-(−)-lupanine followed by chemical transformation of the latter into high added-value D-(+)-sparteine. Thus, the application of biocatalysts for lupanine racemate resolution could substantially reduce the experimentation required for achievement of a cost-effective process.

**Table 1:** Biocatalytic resolution of racemic mixtures

Biocatalyst	Racemic compound	Purified enantiomer	Initial racemate conc. (g L^−1^)	Remaining racemate conc. (%)	ee (%)	References
*P. putida* LPK411	Lupanine	L-(−)-lupanine	1.23	39	99	Current study
*R. rhodochrous* LPK211	Lupanine	L-(−)-lupanine	1.45	60	100	Current study
*Rhodococcus* sp. LPK311	Lupanine	L-(−)-lupanine	1.39	60	96	Current study
*P. putida* (NCIB9494)	Carvedilol	*(S)*-(−)-carvedilol	0.25	34.92	95	Ettireddy et al. [22]
*Rhodococcus* sp. CCZU10-1	Phenyl methyl sulfoxide	*(S)*-phenyl methyl sulfoxide	14.02	55.3	> 99.9	He et al. [23]
*P. putida* (NBRC 14164) ω-transaminase	α-methylbenzylamine	*(R)*-α-methylbenzylamine	6.06	49.8	> 99.0	Wu et al. [24]
	2-amino-2-phenylethanol	*(S)*-2-amino-2-phenylethanol	5.49	49.3	> 99.0	Wu et al. [24]
*R. opacus* L-AAO oxidase	Leucine	d-Leucine	0.91	50	> 99.2	Geueke and Hummel [25]
	Phenylalanine	d-Phenylalanine	1.16	50	> 99.5	Geueke and Hummel [25]

### Biodegradation of lupanine enantiomers by *P. putida* LPK411

**Fig. 2:**
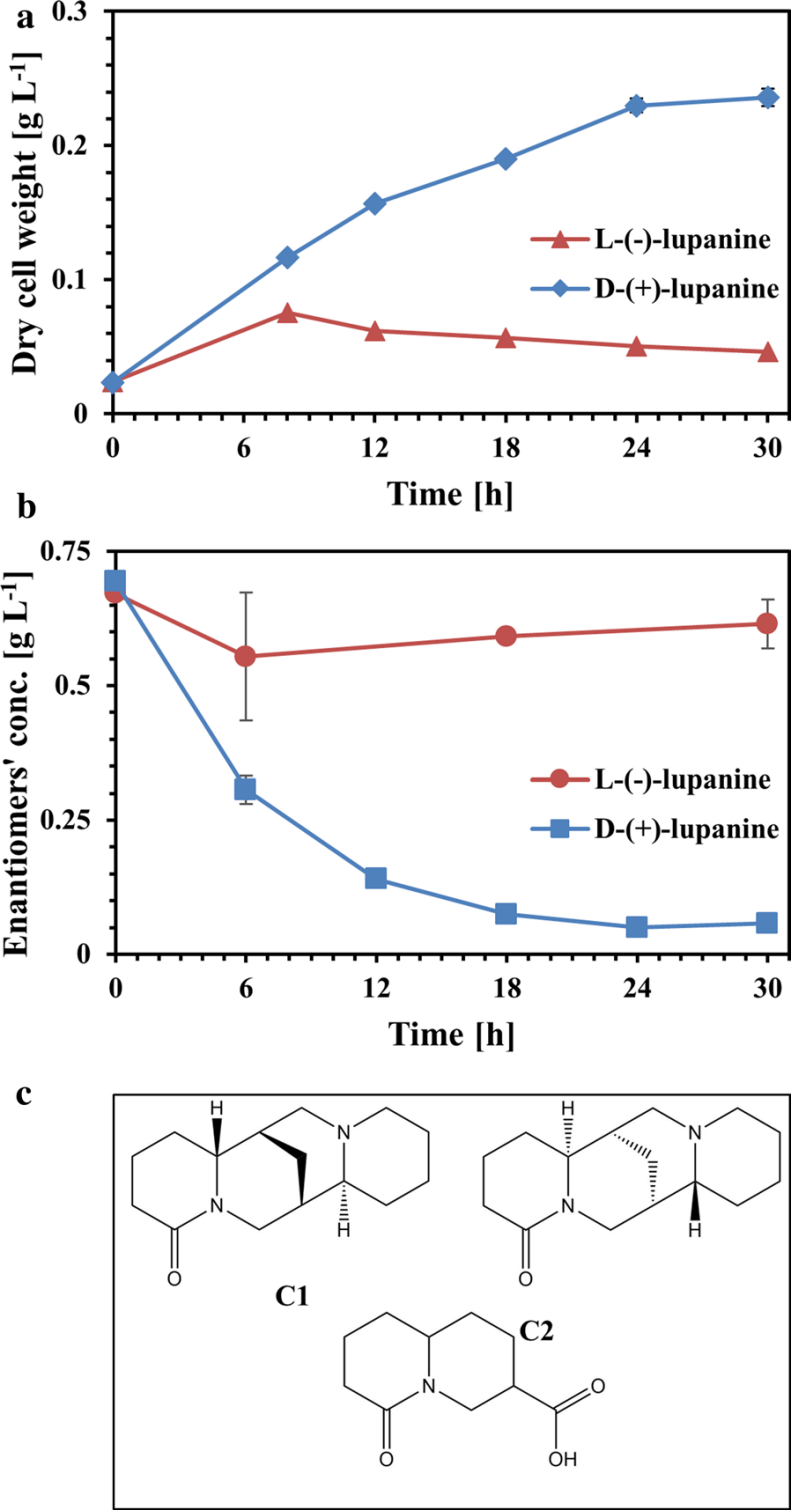
Biodegradation of D-(+)-lupanine and L-(−)-lupanine by *P. putida* LPK411. **a** Biomass growth (dry cell weight concentration), **b** concentration of D-(+)-lupanine and L-(−)-lupanine in fermentations, **c** chemical structure of lupanine enantiomers and end-product formed from D-(+)-lupanine biodegradation. C1: D-(+)-lupanine; C2: L-(−)-lupanine; C3: (trans)-6-oxooctahydro-1H-quinolizine-3-carboxylic acid

The most effective microorganism in performing racemic lupanine resolution was *P. putida* LPK411. Thus, the specific strain was grown in medium containing D-(+)-lupanine and L-(−)-lupanine, which were fed in separate cultures as single substrates (Fig. 2). The results demonstrate that only D-(+)-lupanine could be metabolized substantially by the microorganism. *P. putida* LPK411 achieved D-(+)-lupanine removal of 92% following 30 h of incubation, which resulted in net biomass production of 0.21 g L^−1^. Nevertheless, under the same conditions L-(−)-lupanine biodegradation was insignificant as confirmed by statistical analysis. Moreover, NMR was applied to identify the metabolites generated from D-(+)-lupanine biodegradation by *P. putida* LPK411 following 30 h of incubation. A single compound (molecule C3 in Fig. 2c) was formed as end-product of D-(+)-lupanine biodegradation, which was identified as (trans)-6-oxooctahydro-1H-quinolizine-3-carboxylic acid.

A few studies have focused on biodegradation of racemic quinolizidine alkaloid mixtures, while significantly less attention has been placed in biodegradation of other racemic alkaloids. Hopper and Kaderbhai, [19] suggested that the first step of lupanine biodegradation by *Pseudomonas* sp. occurred through the action of lupanine hydroxylase, an inducible enzyme which was active only when the microorganism was grown on D-(+)-lupanine. The enzyme remained inactive in the presence of L-(−)-lupanine indicating that it could be induced only by growth of the strain on D-(+)-lupanine. Santana et al. [17] demonstrated that the molecules 3-hydroxylupanine, 13-hydroxylupanine, 17-oxosparteine, 3,4-dehydrolupanine and α-isolupanine can be produced during lupanine bioconversion by unidentified microorganisms. Moreover, bioconversion experiments of racemic lupanine using LPK111 and LPK311 generated the same compound (lupanine N-oxide) as final metabolic product [15]. However, the use of *P. putida* LPK411 resulted in accumulation of three metabolic products from racemic lupanine biodegradation (17-oxolupanine and two derivatives incorporating open ring structures). The final metabolic product of D-(+)-lupanine biodegradation by *P. putida* LPK411 identified in the current study has not been previously reported as a metabolite from microbial cultivation on lupanine. However, the presence of the amide and carboxyl functionalities opens the possibility for further chemoselective functional group modifications. Thus, further research is needed to assess the complete metabolic pathway followed by LPK411 for D-(+)-lupanine biodegradation and the potential use of the specific molecule as starting material for chemical synthesis of other valuable products [27].

### Transcriptional kinetics of *luh* gene in *P. putida* LPK411

An increased research interest over the past few years in understanding and optimizing the production of added-value commodities by microbial producers has stimulated various studies pertinent to the metabolism of quinolizidine alkaloids, including lupanine and sparteine [18, 19]. Determination of transcriptional kinetics in microorganisms enables obtaining important information pertinent to the expression of catabolic genes improving the knowledge about waste streams bioconversion to high added-value chemicals and the biodegradation process triggered [28]. Moreover, future optimization of the bioprocess is enabled through the development of coupled gene expression-growth kinetic models [29, 30].

**Fig. 3:**
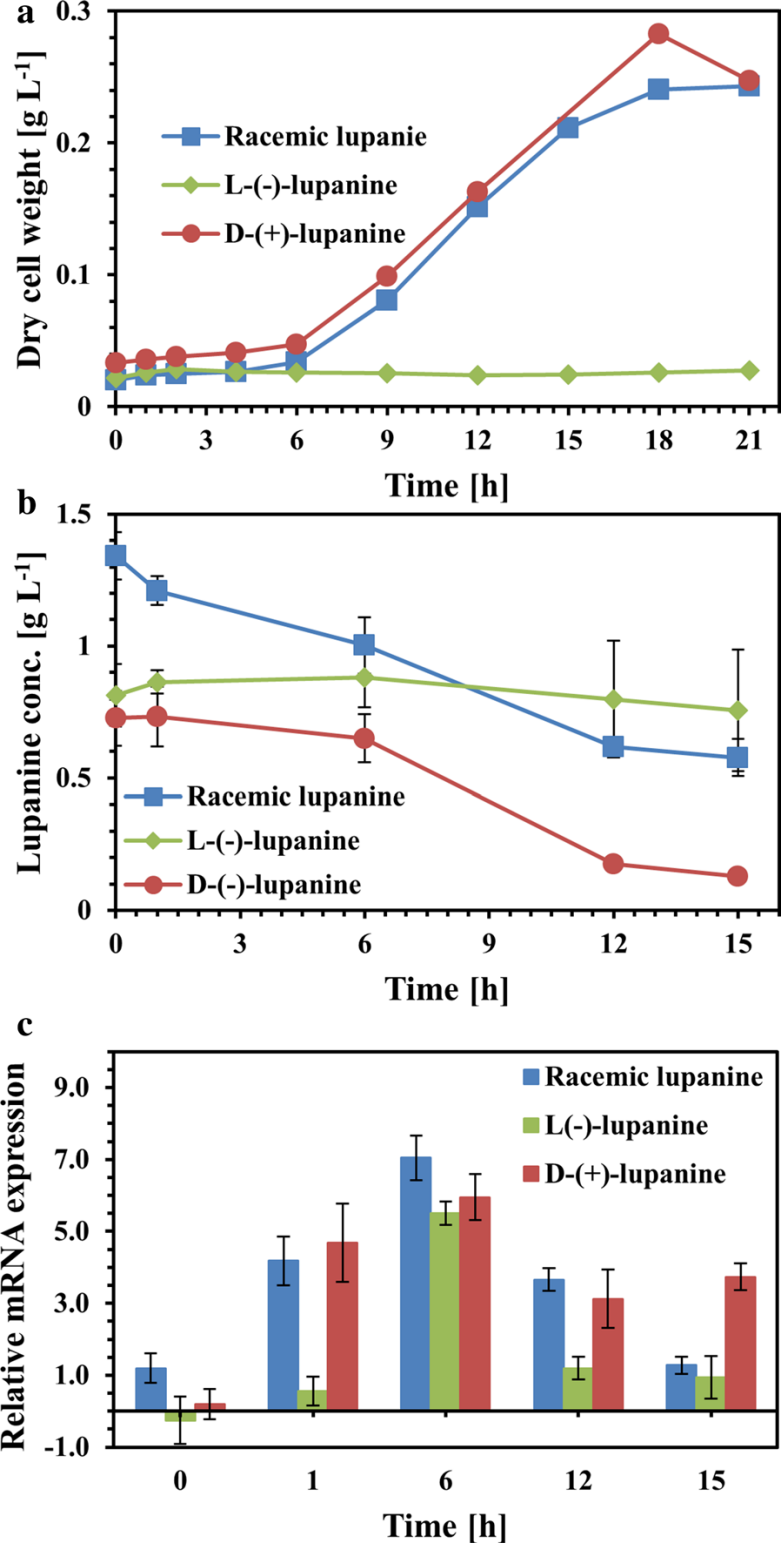
Expression from the *luh* gene of *P. putida* LPK411 during biodegradation of racemic lupanine and lupanine enantiomers. **a** Biomass growth (dry cell weight), **b** concentration of racemic lupanine and lupanine enantiomers and **c***luh* gene relative mRNA expression

The first step in racemic lupanine biodegradation by *Pseudomonas* sp. was performed via the action of the *luh* gene, which encodes for the lupanine hydroxylase enzyme responsible for the conversion of lupanine to 17-hydroxylupanine [19, 31]. Thus, *luh* could be the first gene expressed in the catabolic pathway of lupanine used by LPK411. Herein, systematic monitoring of transcription from the *luh* gene was performed during the course of *P. putida* LPK411 growth in medium containing the racemic alkaloid mixture or lupanine enantiomers (D-(+)-lupanine, L-(−)-lupanine) until biomass reached the stationary phase (Fig. 3). mRNA expression of *luh* was at basal level upon racemic lupanine entry, while at the first stages of the culture *luh* transcription continuously increased (p < 0.05) reaching a peak of expression at 6 h. The direct decrease of lupanine concentration observed since the beginning of the culture supported this claim. Thus, the maximal expression of *luh* occurred at the onset of exponential growth, where racemic lupanine biodegradation had already started, suggesting complete activation of the lupanine biodegradation pathway. Subsequently, *luh* mRNA expression was reduced to basal levels between 12 and 15 h (p < 0.05), where racemic lupanine removal reached 57%. Similarly to racemic lupanine biodegradation, the addition of D-(+)-lupanine triggered the same response of *luh* transcription demonstrating similar mRNA expression pattern (p < 0.05). The only difference was identified at 15 h of fermentation where *luh* expression due to D-(+)-lupanine catabolism was significantly higher as compared to expression triggered by racemic lupanine. Interestingly, *luh* was not expressed immediately following L-(−)-lupanine addition remaining at basal level for the first h of substrate induction. However, expression of *luh* sharply increased following 6 h of enantiomer addition exhibiting similar mRNA level as compared to the use of racemic and D-(+)-lupanine (p < 0.05). Although expression from the catabolic gene was activated with the use of L-(−)-lupanine, which was not biodegraded in the experiment, *luh* mRNA was decreased to basal level for the rest of the culture.

The response of *luh* upon exposure to lupanine enantiomers and the racemic mixture indicates stimulation of the gene by all the molecules used. However, although these results confirm production of the precursor mRNA molecules required for lupanine hydroxylase formation, which catalyses lupanine bioconversion to the first intermediate of the metabolic pathway, L-(−)-lupanine was not biodegraded. Thus, given that the gene is transcribed in the presence of L-(−)-lupanine either the translation process or the activation of lupanine hydroxylase cannot proceed due to the environmental signal preventing its biodegradation. Moreover, the delay of *luh* activation in the culture conducted using L-(−)-lupanine as compared to the cultures supplemented with D-(+)-lupanine and the racemate indicate that an alternative gene activation mechanism could be potentially followed in the presence of L-(−)-lupanine.

A limited number of publications have focused on the microbial metabolism of quanolizidine alkaloids. Detheridge et al. [18] demonstrated that the initial step in lupanine and sparteine degradation was performed through hydroxylation on carbon C-17 by hydroxylase and dehydrogenase enzymes respectively. Moreover, lupanine hydroxylase was active in *P. putida* only when D-(+)-lupanine was supplemented [19]. Previous studies exhibited that lupanine dehydrogenase from *P. lupanini* is a stereospecific enzyme which catalyses only D-(+)-lupanine [32, 33]. According to Adamu et al. [34] stereospecific/stereoselective enzymes recognize and bind to a specific enantiomeric substrate. The present study further supports this claim through monitoring of *luh* transcriptional kinetics, confirming for the first time that *luh* mRNA gene expression is triggered by the racemate and both lupanine enantiomers, which supports the potential stereospecificity of lupanine catabolic enzymes. Nevertheless, further research is necessary to decipher the molecular events that control the catabolic route of the specific alkaloid.

### Toxicological assessment of lupanine enantiomers with marine bacteria

**Fig. 4:**
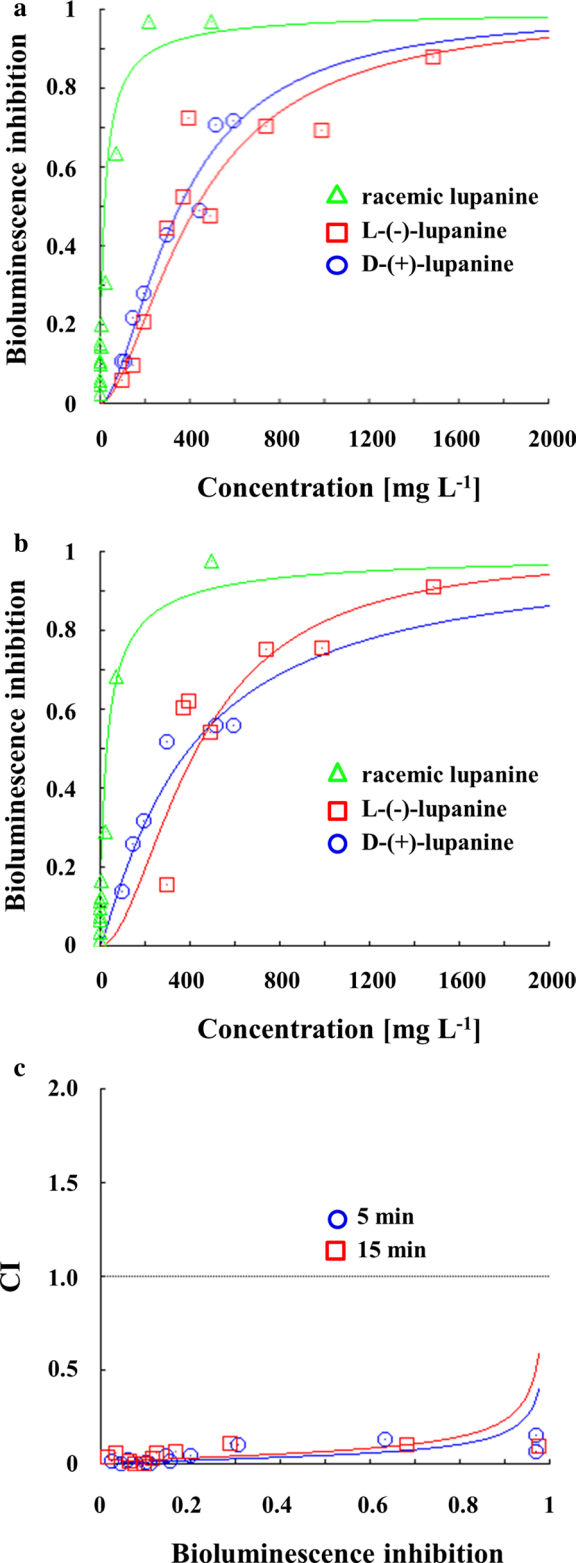
Ecotoxicological assessment of racemate and lupanine enantiomers on *Α. fischeri*. **a** Bioluminescence inhibition for 5 min of exposure, **b** bioluminescence inhibition for 15 min of exposure and **c** toxicity interaction (expressed as CI) values calculated for different durations of exposure to the alkaloid

The marine bacterial strain *A. fischeri* constitutes a microorganism that is commonly employed in microscale toxicity testing, based on its natural bioluminescence capacity. Therefore, the particular ecotoxicological bioassay was applied to assess the acute toxicity of each lupanine enantiomer. The toxicity imposed on the aquatic microorganism was calculated as the EC_50_ value corresponding to two distinct durations of exposure to each enantiomer (Additional file 1: Fig. S1). Τhe toxicological assessment demonstrates that both D-(+)-lupanine and L-(−)-lupanine induced similar levels of toxicity for *Α. fischeri*, based on EC_50_ values obtained for 5 min and 15 min of exposure that ranged between 317–320 mg L^−1^ and 372–457 mg L^−1^ respectively. Moreover, the enantiomers interaction assessment (Fig. 4) demonstrates a very strong synergistic effect of D-(+)-lupanine and L-(−)-lupanine mixture toxicity for *Α. fischeri* (CI < 1).

Previous studies on intravenous administration of coniine, ammodendrine and *N*-methylammodendrine enantiomers in mice demonstrated that the two molecules can incorporate similar LD_50_ values, which could be similar to that of the racemic mixture (Table 2). Moreover, the toxicity of the racemate often constitutes the additive result of both enantiomers, demonstrating an effect that ranges between the toxicity values of the respective enantiomers [35]. However, ecotoxicological studies in mice report that racemic anabasine expressed higher toxicity as compared to the enantiomers [36]. The EC_50_ values calculated for racemic lupanine on *Α. fischeri* have been previously determined for 5 min and 15 min of exposure as 89 mg L^−1^ and 47 mg L^−1^ respectively [15]. Thus, the results presented here indicate that lupanine enantiomers are less toxic as compared to racemic lupanine, confirming the presence of a strong synergistic effect in the toxicity action of these molecules when they exist in the form of a racemic mixture. This response is aligned with a potential absence of a specific mechanism for detoxification/transformation of these compounds in the metabolism of *A. fischeri*, that could result in at least additive effect on bioluminescence inhibition following exposure to the racemic mixture. Lin et al. [37] reported that enantioselective biological reactions, such as in vivo chiral inversion or enantiomerization, could cause antagonistic or synergistic interactions. Nevertheless, only a few studies have yet attempted to understand the interactions between the enantiomers of chiral molecules.

**Table 2:** Ecotoxicological effects of racemic mixtures and the respective enantiomers in different organisms

Compound	Species	Test	Concentration			Reference
			Racemate	(+), *(R)*, D	(−), *(S)*, L	
Lupanine	*A. fischeri*	EC_50_ (5 min)	89 mg L^−1^	320 mg L^−1^	317 mg L^−1^	Parmaki et al. [15], Current study
	*A. fischeri*	EC_50_ (15 min)	47 mg L^−1^	457 mg L^−1^	372 mg L^−1^	Parmaki et al. [15], Current study
Coniine	Mouse	LD_50_ (iv)	7.7 mg kg^−1^	12.1 mg kg^−1^	7.0 mg kg^−1^	Lee et al. [38]
Ammodendrine	Mouse	LD_50_ (iv)	134 mg kg^−1^	94.1 mg kg^−1^	115 mg kg^−1^	Green et al. [36]
Anabasine	Mouse	LD_50_ (iv)	1.6 mg kg^−1^	11 mg kg^−1^	16 mg kg^−1^	Green et al. [36]
*N*-methylammodendrine	Mouse	LD_50_ (iv)	–	56.3 mg kg^−1^	63.4 mg kg^−1^	Lee et al. [39]

*iv* intravenous administration

## Conclusions

Biocatalytic resolution of the lupanine racemate existing in alkaloid rich wastewater could provide an innovative technology for production of useful molecules improving the sustainability of current chemical methods and decrease the ecotoxicological effect of the specific effluent. The microorganisms employed achieved high L-(−)-lupanine excess and the enantioselectivity of LPK411 for D-(+)-lupanine was shown in fermentations through measurements of macroscopic process parameters and via monitoring the transcriptional kinetics of *luh* aiming to decipher the molecular interactions responsible for lupanine enantioselective catabolism. The data obtained hold great potential for development of an environmental friendly biological chiral separation process of lupanine, which could be combined with chemical modification methods to produce a range of added-value molecules.

## Materials and methods

### Purification of lupanine racemate and enantiomers

Racemic and enantiopure lupanine was obtained following a procedure previously described [13, 15]. The purity of lupanine isolated from the above processes was also determined by ^1^H NMR spectroscopy. There were no detectable differences between the respective protons of D-(+)-lupanine and L-(−)-lupanine isomers, since they produce identical chemical shifts. However, impurities assigned to multiflorine were detected in the racemic mixture of lupanine up to 7%, whereas in each specific isolated enantiomeric product D-(+)-lupanine or L-(−)-lupanine, isomer impurities assigned to various quinolizidine derivatives based on the integrals of the protons bound to the unsaturated carbons were quantified up to 4%.

### Microorganisms and culture conditions

All microorganisms used were isolated as presented in Parmaki et al. [15] and they were maintained as glycerol stock preparations at − 80 °C. Subcultures of *P. putida* LPK411 and two *Rhodococcus* strains (*R. rhodochrous* LPK211, *Rhodococcus* sp. LPK311) were pregrown at 31 °C in M9 minimal salts medium overnight using 1.5 g L^−1^ of lupanine racemate as carbon source. The inoculum was grown in 250 mL flasks with a working volume of 50 mL under 100 rpm stirring. Duplicate cultures were prepared under sterile conditions with the addition of 5% (v/v) inoculum volume utilizing racemic lupanine as well as pure lupanine enantiomers (D-(+)-lupanine or L-(−)-lupanine) depending on the requirements of each experiment.

Cultures for biocatalytic resolution of lupanine racemate were conducted through application of 1.5 g L^−1^ racemic lupanine in 500 mL flasks (working volume of 300 mL) and 5% (v/v) of inoculum. For biodegradation of lupanine enantiomers 7.5 mL of pregrown cells were inoculated into 500 mL flasks (working volume 150 mL) containing M9 medium with initial D-(+)-lupanine or L-(−)-lupanine concentration of 0.75 g L^−1^.

Subcultures of *P. putida* LPK411 for determination of transcriptional kinetics of *luh* gene were pregrown for 18 h at 31 °C in M9 supplemented with 1.5 g L^−1^ of succinic acid. The experiments were prepared by diluting pregrown cells in M9 containing 1.5 g L^−1^ racemic lupanine or 0.75 g L^−1^ lupanine enantiomers (either D-(+)-lupanine or L-(−)-lupanine). The optical density (600 nm) at the beginning of the experiment was 0.1 measured on a UV/VIS spectrophotometer (JENWAY 7315, Staffordshire, UK), while cultivation was conducted using 1000 mL shake flasks (200 mL working volume). All experiments were performed at 31 °C, 100 rpm and pH 7.

### Acute toxicity assessment of lupanine enantiomers with marine bacteria

Bioluminescence inhibition of the marine bacteria *Aliivibrio fischeri* (ΝRRL Β11177) following exposure to each lupanine enantiomer (D-(+)-lupanine or L-(−)-lupanine) was assessed employing an acute toxicity microscale test as described previously [15]. Several dilutions that varied between 75 and 1000 mg L^−1^ were prepared using stock solutions of each lupanine enantiomer and 2% NaCl of final salinity, while the pH ranged from 7 to 7.5. Lyophilized bacteria were rehydrated with reconstitution solution and maintained at 15 °C throughout the test.

Luminescence of bacteria was measured following exposure to lupanine enantiomers for 0, 5 and 15 min using a Microtox Model 500 analyzer (Newark DE, USA). Triplicate preparations of each experimental run were conducted and the samples collected were analyzed in duplicate. The compound applied as positive control was phenol. The concentration resulting in population bioluminescence inhibition of 50% (EC_50_) was calculated using linear regression of inhibition as previously presented [15].

### Toxicity interaction assessment of lupanine enantiomeric mixture

Combination index (CI) analysis was conducted to quantify the effect of the lupanine binary mixture using CompuSyn software (Combo-Syn Inc., US). CI values calculation was conducted as previously described in Chou, [40]. Toxicity interaction of the mixture was classified as (i) synergism: CI < 1, (ii) additive effect: CI = 1, and (iii) antagonism: CI > 1.

### Preparation and isolation of total RNA, cDNA synthesis and quantitative real-time PCR (q-PCR)

q-PCR analysis was conducted to measure mRNA expression from the *luh* gene during racemic lupanine biodegradation by *P. putida* LPK411. Depending on the cell density 3–4.5 mL samples were collected and centrifuged at 9000 rpm for 30 min at 4 °C. The harvested cell pellet was soaked in liquid nitrogen and subsequently stored at − 80 °C. Total RNA isolation and cDNA synthesis were performed as previously described [41]. According to the threshold cycle values obtained relative mRNA expression was estimated using Eq. 1. cDNA synthesis and qPCR were performed using a SensoQuest labcycler (SensoQuest GmbH, Göttingen, Germany) and qTower^3^G real-time PCR (Analytik Jena, Jena, Germany) respectively. The primer pairs of the *luh* and *rpoN,* which was used as the reference gene, are displayed in additional data (Additional file 1: Table S1). Duplicate experiments were conducted and two samples were analysed for each replicate comprising analysis of 4 samples in each time point.

1 $$\Delta {\text{C}}_{{{\text{T}},luh}} = {\text{C}}_{{{\text{T}},rpoN}} - {\text{C}}_{{{\text{T}},luh}}$$

### Statistical analysis of mRNA expression data

The objective of statistical analysis was to elucidate the relative mRNA expression profile of *luh* gene. Thus, one-way analysis of variance (ANOVA) was conducted and p < 0.05 was defined as acceptable level of significance.

### Analytical methods

#### Dry cell weight

A UV/VIS spectrophotometer (JENWAY 7315, Staffordshire, UK) was used to measure biomass absorbance (600 nm) and dry biomass was determined using a calibration curve of dry cell weight. The calibration was obtained through dilution of a pre-grown culture to obtain different OD values. Biomedium samples of 10 mL were dried (105 °C) and placed at 600 °C for 60 min to combust the organic content. The coefficient of variation calculated for 3 samples was 4.5% at a biomass content of 0.1 g_biomass_ L^−1^.

#### Determination of lupanine concentration

Gas Chromatograph (GC) analysis was performed to access the racemic and lupanine enantiomers content in samples derived from microbial cultures as previously described [15]. All substances applied were analytical grade and purchased from Sigma-Aldrich Company Ltd (UK).

#### Determination of lupanine ee

High Performance Liquid Chromatography (HPLC) was applied to assess the lupanine enantiomeric excess in microbial cultures. Samples were centrifuged at 4500 rpm and filtered through 0.2 μm syringe filters for biomass removal. 15 mL of the filtrate were basified (pH > 11) using 1 M NaOH solution and the alkaloid content was extracted with application of 40 mL of dichloromethane. Drying of the organic phase was performed with anhydrous Na_2_SO_4_ and subsequent concentration was achieved using a Rotavapor R-3 (BUCHI Labortechnik, Switzerland). Concentrate sample was applied to a pipette containing Celite and the alkaloid content was eluted through application of 4–6 mL dichloromethane. The samples were evaporated until dry and dissolved in 100 μL isopropanol, to which 900 μL of *n*-hexane was added. HPLC analysis was performed using a 4.6 mm × 250 mm × 5 μm CHIRALPAK IC column (Chiral Technologies Inc., West Chester, PA, USA) and a 10 mm guard column. The mobile phase constituted 53% *n*-hexane, 22% isopropanol and 25% *n*-hexane with 0.1% of diethylamine. A flow rate of 1 mL min^−1^ was used, while the method was conducted at room temperature and UV detection (230 nm). Analytical grade chemicals were used at all times.

#### Metabolic products’ identification

Nuclear magnetic resonance spectroscopy (NMR) was employed to identify the metabolites formed as end-products of lupanine enantiomers biodegradation as described in Parmaki et al. [15].

## Supplementary information

**Additional file 1: Table S1.** Primers used in quantitative real time PCR. **Fig. S1.** Ecotoxicological assessment of lupanine enantiomers on the aquatic organism *Α. fischeri*. (A) EC_50_ (mg L^−1^) for 5 min and 15 min of exposure to each alkaloid.

## Data Availability

All data generated or analysed during this study are included in this published article and its additional information files.

## Abbreviations

ANOVA: Analysis of variance
CI: Combination index
EC_50_: Half maximal effective concentration
ee: Enantiomeric excess
GC: Gas chromatograph
iv: Intravenous administration
LD_50_: Lethal dose
NMR: Nuclear magnetic resonance
OD: Optical density
q-PCR: Quantitative real-time PCR
