# Supplementary material for: Resolution of alkaloid racemate: a novel microbial approach for the production of enantiopure lupanine via industrial wastewater valorization
Source: Microb Cell Fact. 2020 Mar 14;19:67. doi: 10.1186/s12934-020-01324-1 (PMC7071741; doi:10.1186/s12934-020-01324-1)
Supplement: Supplementary file 1 — Additional file 1: Table S1. Primers used in quantitative real time PCR. Fig. S1. Ecotoxicological assessment of lupanine enantiomers on the aquatic organism Α. fischeri. (A) EC50 (mg L−1) for 5 min and 15 min of exposure to each alkaloid. [file 12934_2020_1324_MOESM1_ESM.docx]

**Additional Material**

**Table S1.** Primers used in quantitative real time PCR

| Gene | Pair of primers | Description | Source |
| --- | --- | --- | --- |
| *luh* | Forward | 5´- TGCTTGCCACGGTTTCAATG-3´ | Current study |
|  | Reverse | 3´-TTTCTGATCGCTATGCCCGC-5´ | Current study |
| *rpoN* | Forward | 5′-TAACGAAACCCTGATGAAGG-3′ | Tsipa et al. [28] |
|  | Reverse | 3′-AATGTCATGCAGTACCAACG-5′ | Tsipa et al. [28] |

**Fig. S1**

**Fig. S1.** Ecotoxicological assessment of lupanine enantiomers on the aquatic organism *Α. fischeri*. (A) EC_50_ (mg L^-1^) for 5 min and 15 min of exposure to each alkaloid.
